# Genetic and Environmental Influences on the Affective Regulation Network: A Prospective Experience Sampling Analysis

**DOI:** 10.3389/fpsyt.2018.00602

**Published:** 2018-11-28

**Authors:** Laila Hasmi, Marjan Drukker, Sinan Guloksuz, Wolfgang Viechtbauer, Evert Thiery, Catherine Derom, Jim van Os

**Affiliations:** ^1^Department of Psychiatry and Psychology, Maastricht University Medical Centre, Maastricht, Netherlands; ^2^Department of Psychiatry, Yale School of Medicine, Yale University, New Haven, CT, United States; ^3^Department of Neurology, Ghent University Hospital, Ghent University, Ghent, Belgium; ^4^Centre of Human Genetics, University Hospitals Leuven, Leuven, Belgium; ^5^Department of Obstetrics and Gynecology, Ghent University Hospital, Ghent, Belgium; ^6^Department of Psychiatry, Brain Centre Rudolf Magnus, University Medical Centre Utrecht, Utrecht, Netherlands; ^7^Department of Psychosis Studies, Institute of Psychiatry, Psychology and Neuroscience, King's College London, King's Health Partners, London, United Kingdom

**Keywords:** affective mental states, emotions, network, time-series, genetic, psychopathology, early environment, childhood trauma

## Abstract

**Background:** The study of networks of affective mental states that play a role in psychopathology may help model the influence of genetic and environmental risks. The aim of the present paper was to examine networks of affective mental states (AMS: “cheerful,” “insecure,” “relaxed,” “anxious,” “irritated,” and “down”) over time, stratified by genetic liability for psychopathology and exposure to environmental risk, using momentary assessment technology.

**Methods:** Momentary AMS, collected using the experience sampling method (ESM) as well as childhood trauma and genetic liability (based on the level of shared genes and psychopathology in the co-twin) were collected in a population-based sample of female-female twin pairs and sisters (585 individuals). Networks were generated using multilevel time-lagged regression analysis, and regression coefficients were compared across three strata of childhood trauma severity and three strata of genetic liability using permutation testing. Regression coefficients were presented as network connections.

**Results:** Visual inspection of network graphs revealed some suggestive changes in the networks with more exposure to either childhood trauma or genetic liability (i.e., stronger reinforcing loops between the three negative AMS anxious, insecure, and down both under higher early environmental, and under higher genetic liability exposure, stronger negative association between AMS of different valences: i.e., between “anxious” at t-1 and “relaxed” at t, “relaxed” at t-1 and “down” at t, under intermediate genetic liability exposure when compared to both networks under low and high genetic liability). Yet, statistical evaluation of differences across exposure strata was inconclusive.

**Conclusions:** Although suggestive of a difference in the emotional dynamic, there was no conclusive evidence that genetic and environmental factors may impact ESM network models of individual AMS.

## Introduction

Traditionally, mental disorders are conceived as categories based on statistical differentiation between symptoms that cluster together, ignoring the underlying causes. Vinogradov and colleagues (1) proposed an associationist model of the symptom dimension of paranoia and suggested that the origins of psychopathology may lie in a network of mental states giving rise to acute phase transitions. Odgers and colleagues showed that these transitions can be modeled as part of a dynamic system; symptoms can be described as “amplifying” when they become more intense with time, “damped” when intensity decreases until going back to the normal state or “stable” when intensity does not change (2). In a recent essay by Kendler and colleagues, mechanisms of psychiatric symptoms were discussed, suggesting they may be productively viewed as “a complex, mutually reinforcing network of causal mechanisms” including genes, environment, and symptoms themselves (3).

The network theory of mental disorders has gained traction as a novel conceptualization of psychopathology, where symptoms—not latent classes underlying symptoms—are studied as active elements interacting with each other in a symptom network. As an example, in a clinically relevant hypothetical scenario, if an individual suffers from sleep loss, this will lead to fatigue, which in turn may give rise to anxiety that may ultimately produce a feedback loop between anxiety and sleep loss, constantly activating all these symptom nodes in the network to develop into a mental-ill state, such as anxiety disorder (4–6). The network approach to psychopathology has become one of the most trending data-driven research fields, producing impactful research output using cross-sectional symptom data, and recently has moved forward including associations over time.

In addition, networks can also be generated using AMS in healthy subjects rather than symptoms of a mental illness. In the network research, these momentary assessed AMS were also called emotions interchangeably. For example, the AMS down is part of the same continuum as the symptom depressed, but severity is far less. Therefore, besides symptom networks, AMS networks are of interest to get insight in the interplay between emotions over time (7). More importantly, the strength of those networks may differ depending on the presence of risk factors, as is the case with risk factors and psychopathology in patient populations (8).

The experience sampling method (ESM) prompts individuals to record their AMS (e.g., feeling cheerful, fearful, energetic, down or relaxed), anomalous experiences (e.g., feeling suspicious, hearing voices, losing control), and context (e.g., minor stressful events, activity, company) after prompts (i.e., beeps or signals emitted via a watch or some device) occurring at unpredictable moments throughout the day (9). Participants' responses to items in the questionnaire are adjectives qualifying the state of mind or symptoms in the moment of the beep, referred to as momentary mental states. The within-subject design with repeated measurements over time allows for the analysis of temporal associations, and has the potential to reveal dynamic mechanisms of mutually impacting mental states that are neglected in cross-sectional, between-subject designs (8, 10).

Numerous studies using this methodology have demonstrated that AMS interact in dynamic relationships (11). For example, insomnia may lead to changes in both positive affect and negative affect the next day (6) and psychotic symptoms as assessed with ESM is associated with clinical severity in patients with psychotic disorder (8, 12). These interactions result in a network of AMS impacting on each other, where the momentarily assessed mental state is represented by a node and the predictive association over time, between an AMS at the previous time point *t-1 (time lag)* and an AMS at the current time point *t*, are represented by a directed arrow. The arrow or edge is also weighted, with the B coefficient expressing the effect size of the predictive associations. For example, an arrow from “relaxed” to “cheerful” weighted at 0.08 would mean that “relaxed” at *t*−*1* predicts “cheerful” at *t* with a B coefficient of 0.08 (4, 13, 14).

The network approach to study the nature of psychopathology may be extended to examine biological mechanisms underlying the interplay between symptoms, assisting in the search for novel treatments (13, 15, 16). It has been hypothesized that genes and environment may act as risk factors for the development of mental disorders by making the structure of an AMS network “risky”; a similar mechanism can be hypothesized for genetic liability (17, 18). For example, genes and environments may affect the strength of the connections (edges) so that a central symptom initiates a cascade of changes in other symptoms, eventually giving rise to a full-blown mental disorder (11, 13, 19, 20). Using intensive time series data, many ESM studies have investigated the effect of genetic and environmental factors on two constructs created by aggregating responses on AMS items: negative affect and positive affect (e.g., cheerful, enthusiastic, satisfied, and energetic for positive affect) (18). A previous study that used structural equation modeling to assess the extent to which genetic and environmental factors contribute to the variability in daily life of those two constructs, showed that 41% of the association between positive affect and negative affect is attributable to genetic factors (21, 22). Thus, it can be hypothesized that not only the sum scores, but also the connections between the individual items may be influenced by genetic factors. In addition, given the fact that psychopathology is comorbid and transdiagnostic, and that genetic liability to psychopathology is shared, to a large degree, between the different mental disorders, the impact of genes on network models may be studied productively using a measure of genetic vulnerability to general psychopathology (23).

Next to genetic factors, various environmental factors have been associated with psychopathology (17). One of the most studied is childhood trauma (8, 24). It is hypothesized that similar to genetic liability, childhood trauma can play a role in networks of AMS in the general population. However, to our knowledge, no previous study using time-intensive intra-individual data has investigated the extent to which both genetic and environmental factors contribute to the connections between moment-to-moment mental states impacting on each other in a network. The present study aimed to investigate the differences in network connectivity and structure between categories of childhood trauma and genetic liability to psychopathology, focussing on six AMS: “cheerful,” “insecure,” “relaxed,” “anxious,” “irritated,” and “down.”

## Methods

### Participants

The study sample was derived from the East Flanders Prospective Twin Study register (25). The EFPTS is a population-based register, prospectively recording all multiple births in Flanders, Belgium, since 1964 (25). The initial sample consisted of 621 female siblings (twin pairs and 45 sisters) (26). The study was approved by the ethics committee of Maastricht University Medical Centre and all participants provided written informed consent. The current analyses are not overlapping with previous work in this sample.

### Measurements

#### Experience sampling method (ESM)

Participants received a wristwatch and a set of self-assessment booklets, one for each day. The wristwatch was programmed to emit a beep at random moments in each of ten 90-min time blocks between 7.30 a.m. and 10.30 p.m. on 5 consecutive days. The semi-random beep design prevents participants from anticipatory behaviors. The procedure has a high self-reported adherence as shown in a previous study (26). After each beep, participants were asked to complete the self-assessment booklet within 15 min. The items collected by ESM consist of around 40 variables indexing thoughts, current context (activity, social context, location), appraisals of the current situation, and affect. The time at which participants indicated they completed the report was compared to the time of the beep, in order to verify whether the participants had completed the form within 15 min (participants were not able to check beep times retrospectively). All reports completed more than 15 min after the signal were excluded from the analysis as earlier work has shown that outside this interval, reports are less reliable and, therefore, less valid (27). Participants with < 17 valid reports (out of 50, i.e., 33%) were excluded. AMS at each beep were rated by participants on 7-point Likert scales ranging from 1 = “not at all” to 7 = “very.” Before starting the main analyses, a subsample of 6 AMS was selected from all available AMS, using two criteria (1) representativeness with respect to valence and arousal (2) variability within subjects.

First, all AMS were labeled as positive or negative (valence) according to a factor analysis of all AMS performed previously, taking into account the multilevel nature of the current sample (28). Accordingly, the items “content,” “cheerful,” and “relaxed” were described as positive AMS, while the items “guilty,” “lonely,” “down,” and “insecure” were indexed as negative AMS. In contrast, the item “irritated” loaded strongly on both valences (28). This first distinction allowed us to further select variables from the entire affective spectrum.

Second, since in general population studies, many negative affect items have floor effects, so that the normality assumption is violated in analyses, to keep models analysable and interpretable, items with strongest floor effects were avoided. Variability was checked, for each of the AMS items described above, by including the current and lagged AMS in an autoregressive model. Subsequently, the proportion of participants with horizontal slopes was calculated per AMS item. A horizontal slope points to floor effects, demonstrating a restriction of range, which can result in a type II error (29).

Finally, we selected AMS with a maximum within-person time-lagged variability, and that represent each quadrant of the four affective domains defined by valence (based on the factor analysis described above) and arousal (30). This choice ensured calculation of associations with genetic and environmental variables across the entire spectrum of affective states. This resulted in the selection of the following AMS: “cheerful” (positive valence, high arousal), “relaxed” (positive valence, low arousal), “irritated” (loading in both the negative and the positive affect dimensions, high arousal), “down” (negative valence, low arousal), “insecure” and “anxious” (negative valence, high arousal).

#### Childhood trauma

Childhood trauma was assessed using the Childhood Trauma Questionnaire short form (CTQ-SF), which is a 25 item version of the Childhood Trauma Questionnaire including items on physical, sexual, and emotional abuse, and physical and emotional neglect, scored on a 5- Likert scale (e.g., “I was maltreated,” “I was beaten often,” “I was abused,” “There was not enough food,” and “I was neglected”) (31, 32). The CTQ-SF is widely used and validated in various languages, including Dutch (32, 33). At the request of the Flemish Twin Register, the four most explicit items concerning sexual and physical abuse were omitted. If necessary items were reversed before generating the sum score. The continuous variable “childhood trauma” reflected the mean score of the 25 CTQ-items. To visualize the effect of childhood trauma on the network, the childhood trauma variable was recoded into 3 categories of severity. Tertiles were used as cut-off points because the CTQ sum score has no official cut-off points and these cut-off points warrant sufficient numbers of subjects per category.

#### Symptom checklist-90-R

The Symptom Checklist-90-R (SCL-90-R), a reliable and valid self-report instrument for screening a range of symptoms occurring in the past week, was used to index the overall severity of psychopathology (34). The SCL-90-R consists of nine subscales (Somatization, Obsessive-compulsive, Interpersonal-sensitivity, Depression, Anxiety, Hostility, Phobic anxiety, Paranoid Ideation, and Psychoticism), covering the entire range of psychopathology. The SCL-90-R was assessed twice within an interval of 6 months. First, scores were averaged per participant. Subsequently, SCL-90-R was dichotomised using the 75th percentile cut off point in order to define genetic liability of the co-twin (see below).

#### Genetic liability to psychopathology

Genetic liability to psychopathology was determined based on the SCL-90, value (i.e., “low” or “high” psychopathology) in the co-twin and zygosity status, consistent with previous work (18, 23, 34, 35). This procedure resulted in three classes of “genetic liability”: participants with co-twins having a low level of psychopathology (the reference category); participants with a dizygotic (DZ) co-twin with a high level of psychopathology (intermediate level of genetic liability for psychopathology) and participants having a monozygotic (MZ) co-twin with a high level of psychopathology (highest level of genetic liability for psychopathology).

### Statistical analysis

All analyses were performed using Stata version 13.0 (36). To take into consideration the hierarchical structure of the data, multilevel (mixed-effects) linear regression models were fitted using the XTMIXED procedure in Stata, considering that level-one units (multiple observations per individual) clustered into level-two units (level of individual twins), that were nested within level-three units (twin pairs).

#### Associations between t-1 AMS and current AMS

Time-lagged variables were used as predictors in the multilevel models (14). Cheerful at time t was predicted by (i) “cheerful,” (ii) “relaxed,” (iii) “irritated,” (iv) “insecure,” (v) “anxious,” and (vi) “down” at t-1 (lag 1). All lagged variables were person mean centered to disentangle within- subject from between- subject effects (37). The same analysis was performed for each of the other AMS at time point t (dependent variable) in six separate models. Thus, the six AMS variables at t were predicted by all the six AMS variables at t-1. All lagged AMS variables were entered simultaneously in the model, as to assess their independent effects. One example of a regression model is:

$$\begin{array}{l} {\text{Cheerful}}_{\text{ijk}}=\left(\text{B}0+{\text{e}}_{\text{ijk}}\right)+\text{B}1*{\text{lag cheerful}}_{\text{ijk}}+\text{B}2*\\ {\text{lag insecure}}_{\text{ijk}}+\text{B}3*{\text{lag relaxed}}_{\text{ijk}}+\text{B}4*{\text{lag anxious}}_{\text{ijk}}+\text{B}5*\\ {\text{lag irritated}}_{\text{ijk}}+\text{B}6*{\text{lag down}}_{\text{ijk}}+\left(\text{B}7+{\text{u}}_{7\text{ijk}}\right)*{\text{time}}_{\text{ijk}} \end{array}$$

Where time is the beep number over days (1–50), the subscript *i* stands for the assessment level, *j* for individuals, *k* for twin pairs and u7_ijk_ for the random slope of time (see below).

As seen above, the B coefficients (B2–B6) are obtained using linear regression analysis. Because the data includes multiple assessments per person and includes twins, we used a regression analysis that can give valid results despite the multilevel structure. The obtained regression coefficients can be interpreted the same way as in regular linear regression analysis. In terms of network analysis, those B coefficients are then used as weights for the time lagged associations between an AMS at a current time point and the AMS at the next time point. The higher the value of the regression coefficient or weights (in term of network language) the higher the chance the two AMS are associated over time and the value of it gives the quantification of the association.

As the time between lagged and current moment has to be contiguous, and all beep moments were in the waking period of the day, *t-1* AMS variables excluded the last beep moment of a day as a lag of the first beep moment the next day. Analyses were performed across 3 strata of childhood trauma as well as across 3 strata of genetic vulnerability.

#### Random slope of time

A time variable (i.e., beep number, counting from 1 to 50) was included in all regression models since a lagged coefficient can be interpreted as an autocorrelation coefficient only if, conditional on all other fixed effects in the model, no systematic trend is present in the data. Because any trend that may be present could differ across participants, a random slope for *time* was added to the models at the individual level, representing the standard procedure for analysis in network research (37).

#### Permutation testing

Mixed-effects models should ideally include random slopes for all time-varying predictor variables (and use fully unstructured covariance matrices for the random effects) (38). This procedure allows for standard errors and thus *p*-values to be correctly estimated. However, this approach is not feasible in the present context, due to the large number of predictor variables and hence the large number of parameters that would need to be estimated (attempts to fit such models result in convergence problems). Therefore, a single random slope for *time* was included in the model (see above), and to obtain valid *p*-values, permutation tests examined the statistical significance of observed B coefficients.

Permutation testing is developed to obtain the distribution of regression coefficients under the null hypothesis. Subsequently, the observed regression coefficient obtained from the real analysis is placed on this normal distribution, to obtain a valid *p*-value. For this, data in which there is no association (the null hypothesis assumption) were analyzed. For example, for the first set of permutations, whose aim is to test the significance of each association between two AMS, i.e., each observable regression coefficient (see below), the dependent variable was removed from the data and shuffled in a random order and merged to the original data, while keeping the multilevel structure. The regression coefficients are calculated repeatedly for 1,000 times using that data to draw a normal, under the null hypothesis, distribution. The percentage of permuted regression coefficients that is to the more extreme end of this distribution than the observed regression coefficient gives the *p*-value (2-sided). The *p*-value is considered significant at the threshold of 0.0162 after Simes correction (0.0002 for between-groups comparison) (see below).

Two different types of permutation tests were performed. The first type was used to obtain valid *p*-values for each regression coefficient (edge weight). The second type was performed to compare regression coefficients across different strata of genetic vulnerability and childhood trauma.

For the first set of permutations, the value of the outcome variable (e.g., “cheerful” at *t*) was removed from each record of the original data file and reassigned to the same participant in random order in a copy of the original data set. Because assessments were shuffled within participants, the level of clustering within the data described above was unchanged. Refitting the model based on the permuted data then provides estimates of the model coefficients under the null hypothesis of no association. By repeating this process, a 1,000 time, a distribution of the regression coefficients under the null hypothesis was generated. Then, the observed coefficients were compared with the respective regression coefficient under the null hypothesis distribution to obtain *p*-values (i.e., the proportion of times that the coefficient in the permuted data was as large as or larger than the observed coefficient; multiplied by two to obtain a two-sided *p*-value). Given 2 × 3 × 6 × 6 tests for statistical significance, Simes correction for multiple testing was applied (39). Graphs derived from the analyses are shown both before and after Simes correction for multiple testing. While main results are the Simes corrected slopes, presentation of the figures with uncorrected slopes prevents conclusions being directly drawn on differences that are merely the result of differences in power related to sample size in subgroups during the calculation of the Simes correction.

In the second set of permutations, the values of the childhood trauma variable were randomly assigned to the participants in another copy of the original data set. Again, regression coefficients in the original data were compared with regression coefficients under the null hypothesis of no difference in regression coefficients between the childhood trauma strata. With this procedure, all regression coefficients of the 36 connections (edges) in the network were tested for differences between the childhood trauma strata, regardless of the level of significance obtained with the first type of permutation testing. This same procedure was repeated for the different strata of genetic liability. Again, Simes correction for multiple testing was applied.

### The construction of AMS networks

The regression coefficients (B1–B6) obtained from the equation in section Associations Between t-1 AMS and Current AMS were represented in a graph to express the bidirectional time lagged associations between each set of two AMS.

A complete set of analyses in one stratum yielded 36 unstandardized regression coefficients (B). These coefficients were represented in a graph using the following procedure:

A 6-by-6 matrix with the regression coefficients (B) was constructed. The connection thus denotes the extent to which the AMS variable (e.g., cheerful) at time point t-1 predicts another AMS variable (e.g., relaxed; **→***B*_*cheerful*−*relaxed*_) at time point t, while controlling for all other variables. The elements on the diagonal are the autoregressive effects (self-loops, e.g., *B*_*cheerful*−*cheerful*_). This procedure was applied in the 3 strata of childhood trauma and the 3 strata of genetic liability, separately (in total 6 graphs). Visualization of networks was obtained using R (qgraph package) (40).

### Assessment of the network structure: centrality indices

Besides quantitative assessment of the connections in the network, another important set of parameters for assessing the influence of genetic and environmental factors on the characteristics of the network are the node centrality indices. Centrality analyses allow for the identification of AMS that are more “central” than others in the network. Given their centrality, they are able, when triggered, to create a “domino effect” and activate the other AMS (13). Three well-known centrality indices were calculated, allowing for a descriptive comparison across the three genetic liability and the three trauma strata: node strength, closeness centrality and betweenness centrality (40, 41). The *node strength or strength* is the sum of the absolute value of the weighted connections (both inward and outward) of a specific AMS, thus indexing the extent to which this AMS is connected in the network. Self-loops (e.g., regression weight between e.g., down at t-1 and down at t) are counted twice as to fulfill the definition of the Strength taking into account the fact that self-loops are good indicators of emotions inertia, previously described as an indicator of increased vulnerability and decreased psychological flexibility (42, 43). *Closeness centrality* is defined as the inverse sum of the shortest distances to all other nodes, where the shortest distances are the sum of the inverse of the regression coefficients. It measures the potential impact of a specific node on each of the included AMS (higher closeness means more impact) (41). *Betweenness centrality* of a node is the number of shortest paths between any two other nodes that pass through that particular node. A node with high betweenness centrality lies on many shortest paths. Thus, a node with a high betweenness centrality means that there is a high number of connections between AMS that depend on that specific AMS, thus increasing its capacity to regulate interactions in the network. More detailed information on these centrality indices can be found elsewhere (13, 41). All indices, except node strength, were computed using qgraph in R (40, 44). Node strength centrality was calculated using the function graph strength in the igraph package in R (44).

## Results

### Sample characteristics

Of the initial study population of 621 individuals, 610 completed the ESM procedure and returned the questionnaires. Twenty-five participants were excluded because of too few valid assessments, leaving a sample of 585 individuals (328 monozygotic twins, 208 dizygotic twins and 45 sisters); the 45 sisters were excluded from the genetic liability analysis (*n* = 540). Participants were aged between 18 and 61 years (mean age 27.7 years; SD 7.9). The larger part of the sample (63.5%) had a college or university degree, 35% had completed secondary education, and 2% had completed primary education only. The majority was in a relationship (75%), and most of the participants were employed (95%).

The average childhood trauma score was 1.66 (SD 0.58), and the average SCL-90-R score was 1.37 (SD 0.33). Mean levels of AMS were as follows: Cheerful 4.63 (1.40); insecure 1.41 (SD 1.02); relaxed 4.77 (SD 1.44); anxious 1.23 (SD 0.76); irritated 1.58 (SD 1.28); down 1.36 (SD 0.96). Table 1 presents the sample characteristics and AMS levels stratified by childhood trauma and genetic liability.

**Table 1:** Descriptives stratified by childhood trauma and genetic liability.

	**Low**	**Medium**	**High**
**CHILDHOOD TRAUMA**			
N (subject level)	190	193	192
Mean age	26.2 (*SD* = 7.07)	26.4 (*SD* = 7.57)	30.7 (*SD* = 8.29)
Range	18–46	18–58	18–61
Mean Trauma Score	1.19 (*SD* = 0.11)	1.51 (*SD* = 0.09)	2.29 (*SD* = 0.58)
n (assessment level)	6992	7072	6786
Cheerful (mean)	4.81 (*SD* = 0.88)	4.68 (*SD* = 0.81)	4.39 (*SD* = 0.83)
Insecure (mean)	1.29 (*SD* = 0.43)	1.43 (*SD* = 0.56)	1.51 (*SD* = 0.67)
Relaxed (mean)	4.96 (*SD* = 0.83)	4.84 (*SD* = 0.81)	4.51 (*SD* = 0.76)
Anxious (mean)	1.14 (*SD* = 0.24)	1.24 (*SD* = 0.37)	1.30 (*SD* = 0.48)
Irritated (mean)	1.38 (*SD* = 0.47)	1.55 (*SD* = 0.60)	1.81 (*SD* = 0.73)
Down (mean)	1.20 (*SD* = 0.37)	1.38 (*SD* = 0.50)	1.51 (*SD* = 0.65)
	**Low liability**	**High liability in DZ**	**High liability in MZ**
**GENETIC LIABILITY**			
N (subject level)	390	54	77
Mean age	27.2 (*SD* = 7.16)	27.99 (*SD* = 8.72)	26.11 (*SD* = 7.24)
Range	18–46	18–46	18–44
Mean Scl-90 total score in the co-twin	1.22 (*SD* = 0.13)	1.85 (*SD* = 0.35)	1.83 (*SD* = 0.32)
n (assessment level)	14342	1873	2897
Cheerful (mean)	4.70 (*SD* = 0.84)	4.35(*SD* = 0.90)	4.49 (*SD* = 0.87)
Insecure (mean)	1.34 (*SD* = 0.46)	1.66 (*SD* = 0.77)	1.54 (*SD* = 0.68)
Relaxed (mean)	4.85 (*SD* = 0.77)	4.54 (*SD* = 0.86)	4.61 (*SD* = 0.96)
Anxious (mean)	1.19 (*SD* = 0.32)	1.33(*SD* = 0.40)	1.33 (*SD* = 0.53)
Irritated (mean)	1.52 (*SD* = 0.59)	1.81 (*SD* = 0.77)	1.72 (*SD* = 0.69)
Down (mean)	1.29 (*SD* = 0.45)	1.47 (*SD* = 0.66)	1.59 (*SD* = 0.70)

*SD, Standard deviation; DZ, Dizygotic twins; MZ, Monozygotic twins; Scl-90, Symptoms checklist-90*.

### Environmental effects in the affective regulation network

**Figure 1:**
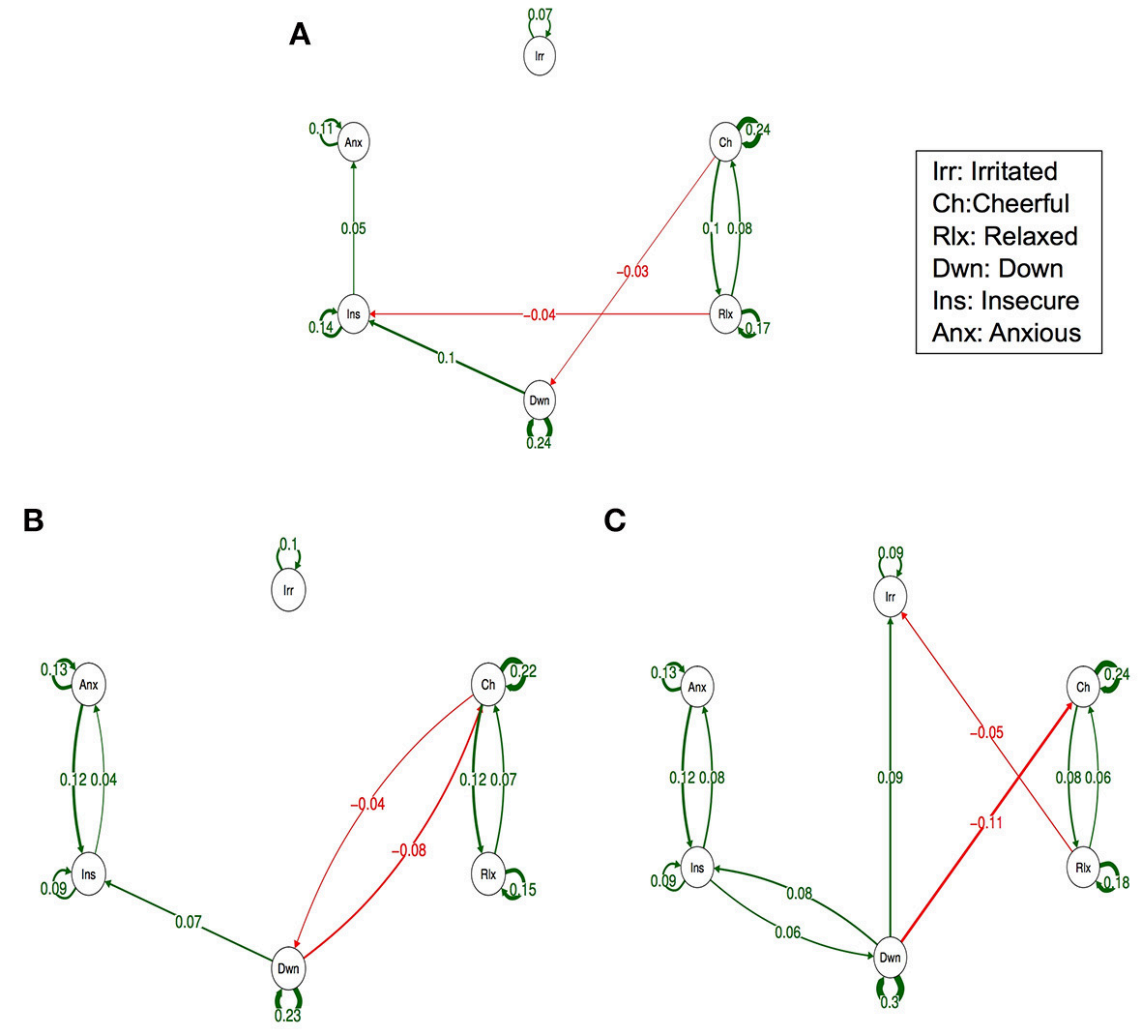
Networks of momentary affective mental states (AMS) in subjects with low **(A)**, Medium **(B)** and high exposure to childhood trauma **(C)**. In this figure, the arrows represent associations over time, i.e., the B coefficient expressing the effect size of the predictive associations. For example, in the low childhood trauma network, there is an arrow from “relaxed” to “cheerful,” meaning that “relaxed” at t−1 predicts “cheerful” at t with a B coefficient of 0.08. Green arrows represent positive associations, and red arrows represent negative associations. The linewidth represents the strength of the association and is determined by the regression weights: the wider the line, the stronger the association (and vice versa). Only significant associations after Simes correction for multiple testing are displayed (alpha is 0.0162).

Figure 1 depicts the dynamic network structure corrected for multiple testing between the six AMS in each of the three childhood trauma exposure groups. When applying Simes correction for multiple testing, alpha was 0.0162. Whereas, the corrected alpha for comparing *p*-values between groups was 0.0002 (Table 2). For the complete network structure see Supplementary Figure 1.

**Table 2:** Comparison between regression coefficients in the different childhood trauma strata (*p*-values presented were obtained from permutation tests of between group differences, Simes corrected alpha = 0.0002).

	**Cheerful_t_**	**Insecure_t_**	**Relaxed_t_**	**Anxious_t_**	**Irritated_t_**	**Down_t_**
**MEDIUM VS. LOW LEVEL OF CHILDHOOD TRAUMA**						
Cheerful_t−1_	0.45	0.28	0.57	0.95	0.72	0.66
Insecure_t−1_	0.41	0.22	0.38	0.92	0.61	0.75
Relaxed_t−1_	0.82	0.39	0.55	0.6	0.67	0.39
Anxious_t−1_	0.88	0.1	0.58	0.7	0.54	0.67
Irritated_t−1_	0.57	0.32	0.57	0.56	0.52	0.56
Down_t−1_	0.56	0.45	0.79	0.71	0.87	0.75
**HIGH VS. MEDIUM LEVEL OF CHILDHOOD TRAUMA**						
Cheerful_t−1_	0.87	0.39	0.61	0.79	0.57	0.78
Insecure_t−1_	0.68	0.19	0.2	0.22	0.9	0.13
Relaxed_t−1_	0.4	0.3	0.74	0.3	0.13	0.81
Anxious_t−1_	0.23	0.17	0.59	0.71	0.18	0.24
Irritated_t−1_	0.5	0.16	0.54	0.91	0.58	0.37
Down_t−1_	0.15	0.6	0.39	0.91	0.35	0.21
**HIGH VS. LOW LEVEL OF CHILDHOOD TRAUMA**						
Cheerful_t−1_	0.53	0.85	0.3	0.81	0.79	0.81
Insecure_t−1_	0.62	0.96	0.68	0.18	0.53	0.24
Relaxed_t−1_	0.52	0.85	0.37	0.59	0.3	0.55
Anxious_t−1_	0.3	0.83	0.95	0.94	0.46	0.12
Irritated_t−1_	0.94	0.71	0.22	0.49	0.95	0.76
Down_t−1_	0.38	0.84	0.52	0.62	0.44	0.11

When visually inspecting the figures, the edges between insecure and anxious seem stronger in the strata under higher childhood trauma exposure with significant reinforcing loops between the three negative AMS; anxious, insecure, and down. However, differences in edges strength between the levels of trauma were not statistically significant (Table 2).

**Figure 2:**
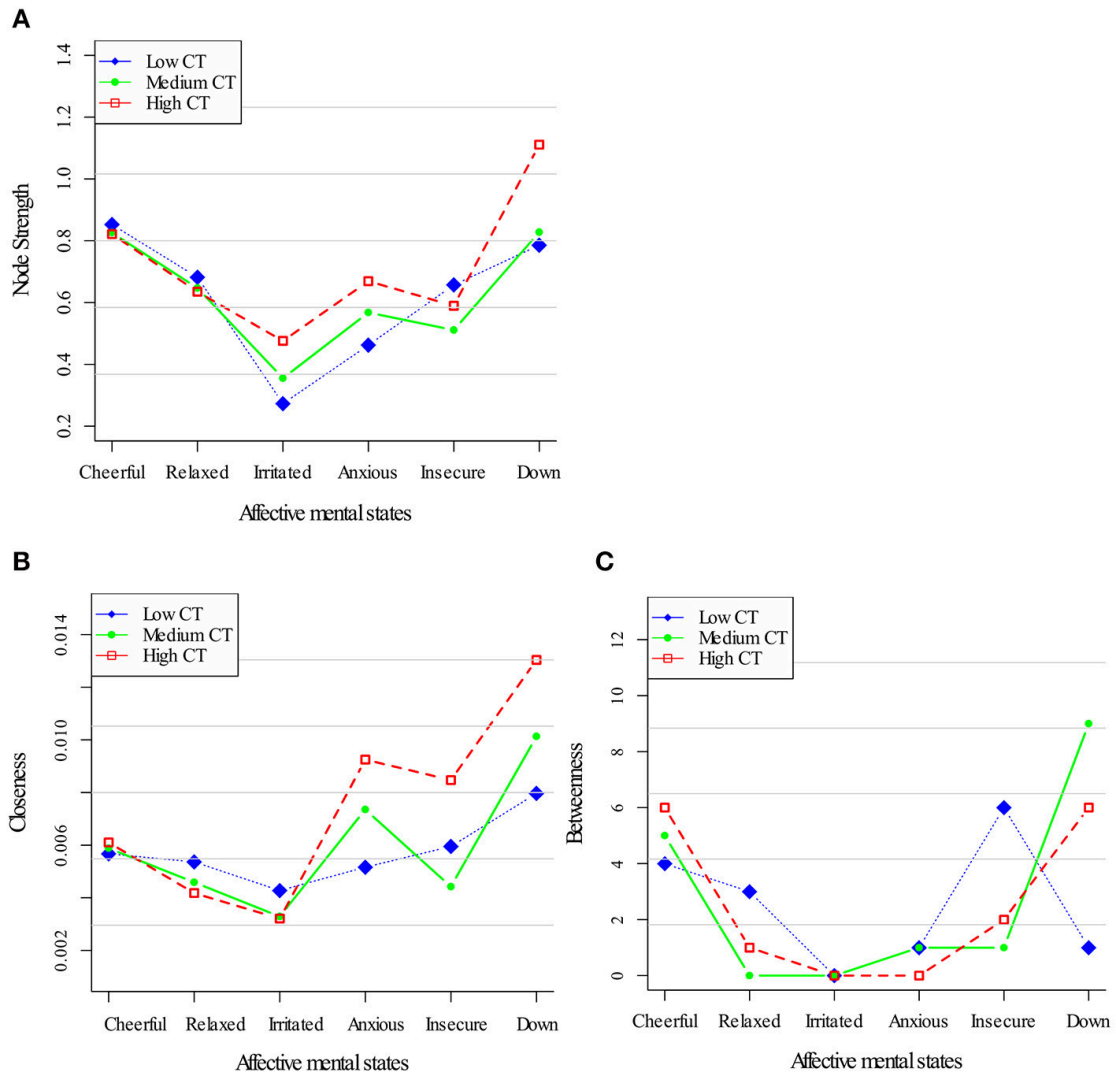
Centrality measures for the childhood trauma exposure networks. Three node centrality measures: Node Strength **(A)**, Closeness **(B)**, and Betweenness **(C)**, of low, medium, and high levels of childhood trauma exposure.

Figure 2 displays centrality indices in the three networks of childhood trauma exposure. In terms of node strength (Figure 2A), a similar pattern for all AMS was apparent. In all childhood trauma strata, closeness centrality as well as node strength was stronger for down (Figure 2B). Although there were differences in both centrality indices between the strata [a profile markedly dominated by the three negative AMS (anxious, down, insecure) in the high childhood trauma network] neither dose-response pattern nor any consistent pattern in the other centrality measures was present. Regarding the Betweenness centrality, the low childhood trauma group, “cheerful” displayed the highest value; while in the high childhood trauma group this was the case for “down” (Figure 2C).

### Genetic effects on the affective regulation network

**Figure 3:**
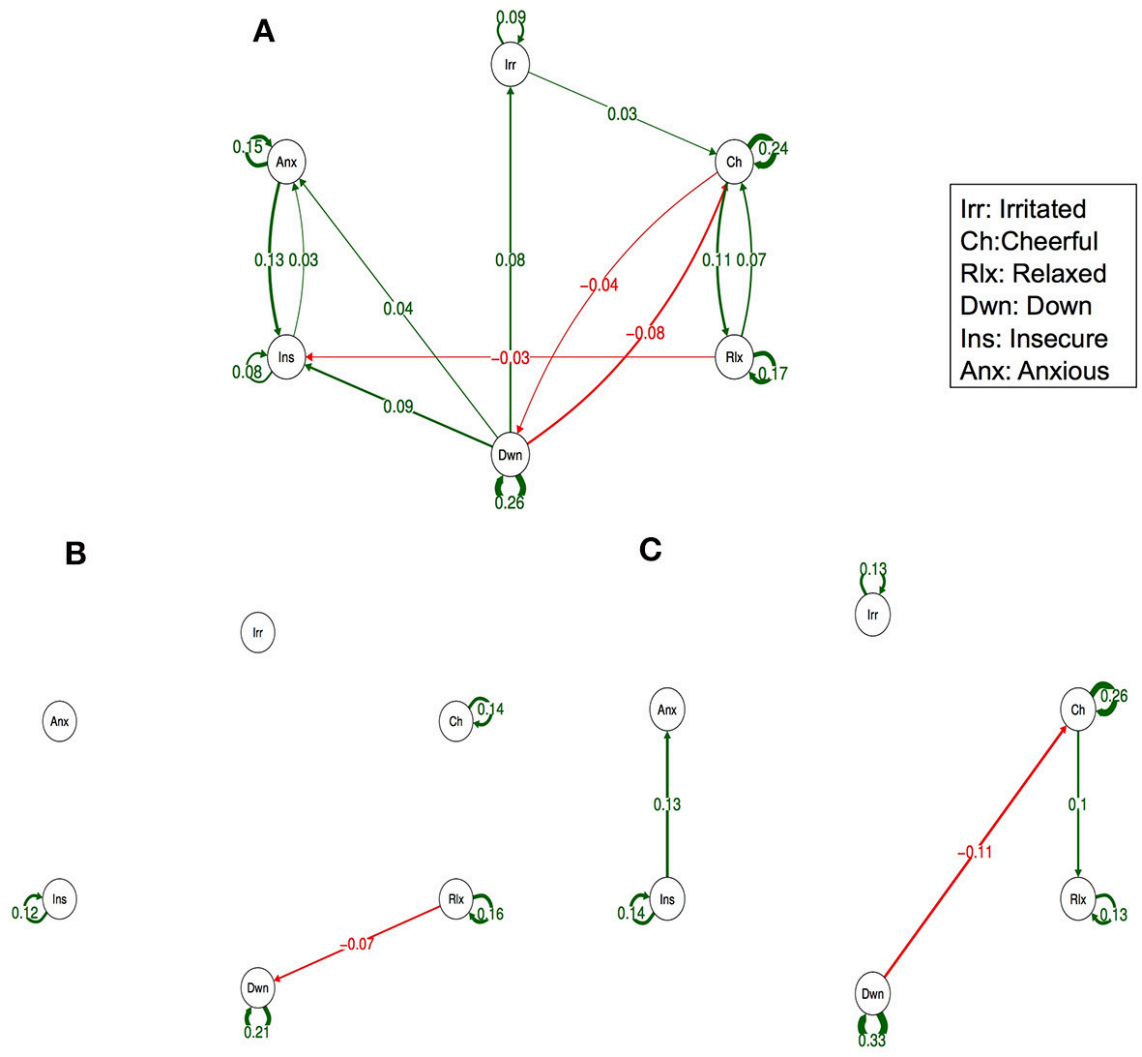
Networks of momentary affective mental states (AMS) in participants with low **(A)**, intermediate **(B)**, and high genetic liability for psychopathology **(C)**. In this figure, the arrows represent associations over time, i.e., the B coefficient expressing the effect size of the predictive associations. For example, in the low genetic liability network, there is an arrow from “relaxed” to “cheerful,” meaning that “relaxed” at t−1 predicts “cheerful” at t with a B coefficient of 0.07. Green arrows represent positive associations, and red arrows represent negative associations. The line width represents the strength of the association and is determined by the regression weights: the wider the line, the stronger the association (and vice versa). Only significant associations after Simes correction for multiple testing are displayed (alpha is 0.0162).

Figure 3 (Simes corrected) and Supplementary Figure 2 (complete network) show the networks stratified by genetic liability. Visual inspection of the complete networks across the three strata of genetic liability indicate that, the loops between “anxious,” “insecure,” and “down” were stronger in the subgroup under higher genetic exposure when comparing it to the one under a low exposure. Additionally, the network in the intermediate liability subgroup was most different with stronger negative association between AMS of different valences, i.a. between “anxious” at t-1 and “relaxed” at t, between “relaxed” at t-1 and “irritated” at t, and between “relaxed” at t-1 and “down” at t. By statistically testing for significance and after Simes correction of the alpha (alpha = 0.0162), only the connection between “relaxed” at t-1 and “down” at t remained significant, and none of the differences between the strata were statistically significant (alpha = 0.0002, Table 3).

**Table 3:** Comparison between regression coefficients in the three genetic liability strata (*p*-values presented were obtained from permutation tests of between group differences, Simes corrected alpha = 0.0002).

	**Cheerful_t_**	**Insecure_t_**	**Relaxed_t_**	**Anxious_t_**	**Irritated_t_**	**Down_t_**
**INTERMEDIATE LIABILITY VS. LOW**						
Cheerful_t−1_	0.07	0.9	0.4	0.23	0.56	0.31
Insecure_t−1_	0.68	0.38	0.91	0.44	0.25	0.60
Relaxed_t−1_	0.69	0.78	0.91	0.61	0.07	0.02
Anxious_t−1_	0.98	0.17	0.56	0.6	0.86	0.90
Irritated_t−1_	0.8	0.56	0.18	0.07	0.1	0.28
Down_t−1_	0.84	0.91	0.55	0.91	0.23	0.48
**HIGH LIABILITY VS. LOW**						
Cheerful_t−1_	0.48	0.7	0.79	0.64	0.82	0.9
Insecure_t−1_	0.16	0.16	0.35	0.01	0.95	0.4
Relaxed_t−1_	0.13	0.19	0.34	0.12	0.72	0.26
Anxious_t−1_	0.11	0.64	0.37	0.32	0.72	0.84
Irritated_t−1_	0.41	0.52	0.58	0.85	0.45	0.46
Down_t−1_	0.6	0.85	0.81	0.95	0.88	0.21
**INTERMEDIATE LIABILITY VS. HIGH**						
Cheerful_t−1_	0.04	0.67	0.6	0.17	0.73	0.34
Insecure_t−1_	0.54	0.85	0.44	0.23	0.32	0.84
Relaxed_t−1_	0.16	0.25	0.62	0.16	0.24	0^*^
Anxious_t−1_	0.25	0.41	0.26	0.8	0.69	0.9
Irritated_t−1_	0.73	0.35	0.46	0.09	0.08	0.12
Down_t−1_	0.86	0.98	0.69	1.06	0.28	0.15

* *p < 0.0002*.

**Figure 4:**
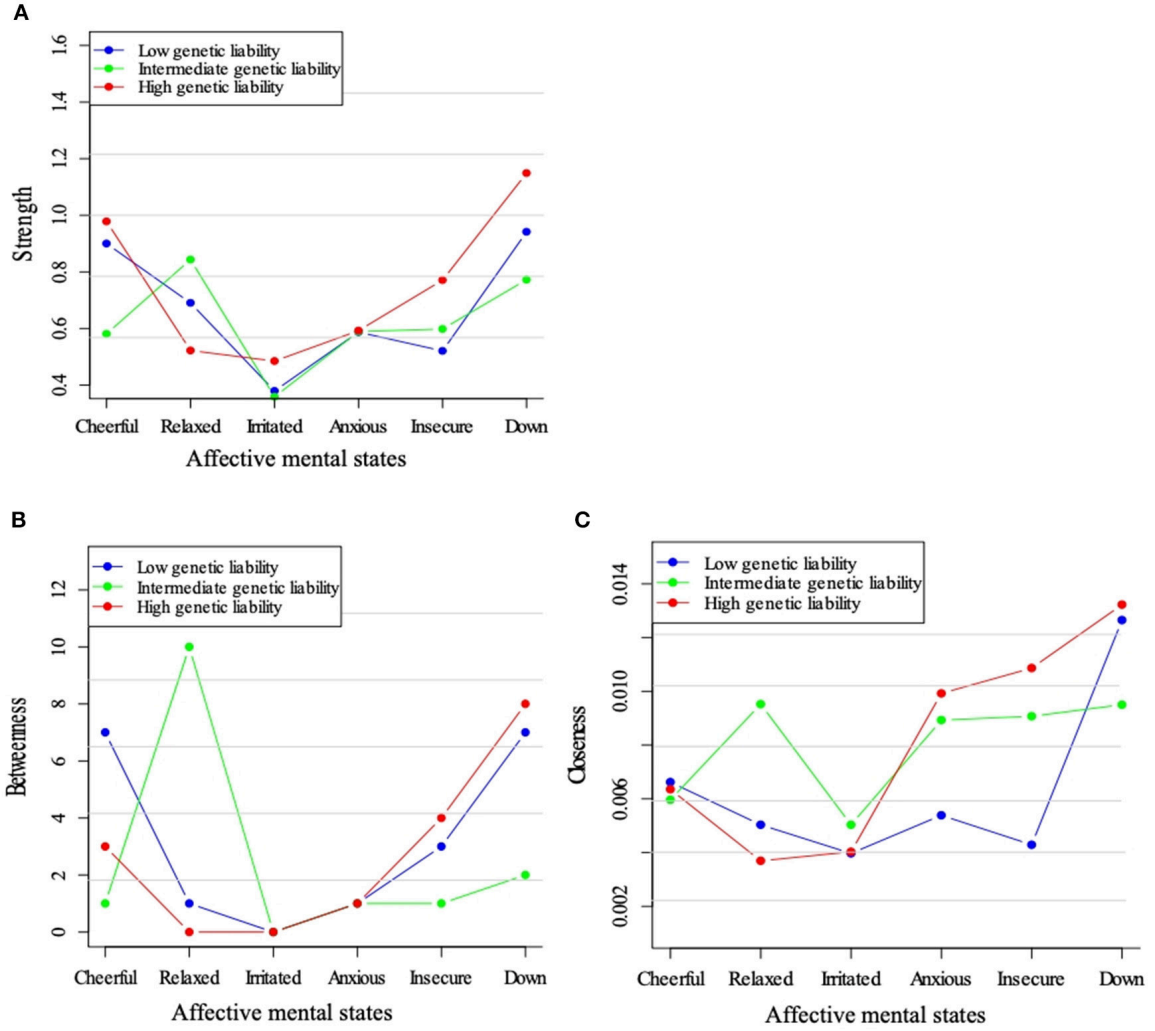
Centrality measures for the networks across levels of genetic liability for psychopathology. Three node centrality measures: Strength **(A)**, Betweenness **(B)**, and Closeness **(C)**, of low, intermediate, and high genetic liability for psychopathology networks.

Figure 4 shows the centrality indices for the three genetic liability subgroups. Feeling “down” displayed a high node strength in all three strata and strength with a maximum in the high liability group (Figure 4A). The positive mental state “relaxed” appeared to play a central position in the intermediate liability group, only. When visually checking betweenness, this same pattern was visible. Closeness centrality was also high for “down” in all three strata (Figures 4B,C).

## Discussion

We investigated the effect of genetic and environmental factors at the level of momentarily assessed AMS in daily life, from a dynamic network perspective. An initial objective of the study was to study differences in networks between strata of childhood trauma and genetic liability, when the networks included six AMS: “cheerful,” “insecure,” “relaxed,” “anxious,” “irritated,” and “down.” Across different levels of trauma or with increasing genetic liability, we expected increased strength of the dynamical associations between AMS as was reported previously (34). However, this previous study assessed strata of symptom severity as opposed to the present study that assessed strata of genetic liability and childhood trauma. In the present study, differences in strength were globally inconsistent and non-significant.

Visual inspection of the networks stratified by childhood trauma showed small differences in the direction of more reinforcement between negative AMS. The differences between the intermediate genetic liability network (i.e., high psychopathology in dizygotic co-twin) and the network in the other two genetic strata seemed larger (in the graphs including all slopes as well as in the graphs including Simes corrected slopes, only). In addition, the small number of subjects in some of the genetic liability strata may have contributed to the observation that some findings did not survive Simes correction. For a more global overview of complete networks we refer to Supplementary Figures 1, 2.

Network representations of momentary psychopathology in the ESM paradigm statistically may have low sensitivity in identifying and quantifying effects of childhood trauma or genetics, especially since many previous studies demonstrated specific molecular genetic significant associations with emotion dynamic parameters. Among them was the recent positive finding suggesting the link of the serotonin transporter gene polymorphism (5-HTTLPR) to emotional inertia of negative emotions applying the ESM methodology in collecting data (45). Yet, this study used sum scores of emotions, i.e., negative affect, and positive affect, in which the possible intrinsic dynamic between individual negative or positive AMS exposed in the present paper would have been collapsed, and therefore blinded, to give emotional inertia overtime. Both approaches, using individual AMS and sum scores, might be complementary in future studies. Alternatively, however, it is possible that the combined impact of interacting environmental and genetic factors on emotion dynamics, as captured by ESM, may yield highly person-specific patterns of variation, making it more difficult to identify patterns that are valid at the level of the group and between groups. Another, related, reason for the lack of significant findings may be that ESM ratings of e.g., “insecure” and “relaxed” may have low reliability. Sum scores of related ESM items may be more reliable than individual items (46). Finally, the fact that directionally visible differences remained statistically inconclusive across levels of genetic and childhood trauma exposure may be inherent to the low power of permutations tests in the context of the ESM network analysis.

### Strengths and limitations

An important strength of the current study is that it used a large number of observations due to the nature of ESM methodology. This allowed us to compare three groups across both environmental and genetic exposures. Cross-sectional network analysis can be seen as an improved factor analysis or principal component analysis, visualizing connections between mental states assessed once over periods of weeks or months, with standard instruments (5). The present paper as well as other recent work (14) generated networks including a time component using ESM data, enabling studying changes in symptom levels over time rather than analyzing a summarized measure over a longer period. Despite using a limited set of AMS, networks including a time component again showed the importance of clustering of symptoms. Additionally, it showed that networks are dynamic: clustering of symptoms changes from moment to moment.

It could be argued that childhood trauma can be a consequence of genetic liability because it can be a result of parents with more psychopathology having more problems with child upbringing (gene-environment correlation) (47). However, a cross tabulation between childhood trauma and genetic liability in the present data showed only a mild correlation (in the lowest trauma tertile proportion of high genetic liability (29%) was lower than in the other trauma tertiles (48, 45%). Despite this association most part of the trauma variable can be attributed to other factors than genetic liability and the study of both variables in two different sets of analyses is warranted.

This is the first study using the network methodology in answering an etiological research question involving genetic liability and early environment. As data were initially collected in the general population, some limitations are inherent. While the advantage of a representative sample is that it best captures the natural spectrum of psychopathology, a limitation is that negative affect items were rare and, therefore, not normally distributed. However, because items with high levels of variation were selected to avoid floor effects and because permutation tests (free of distributional assumptions) were performed, it did not lead to invalid methods of analysis. A second limitation was the impossibility to include random effects for the slope of all predictor variables in the model. Therefore, standard methods for testing the model coefficients would have led to invalid *p*-values. However, we have shown that by applying advanced statistical techniques, permutation tests, valid and interpretable results can be obtained. Such an approach may prove useful for other computational network problems; even though statistical power may be negatively affected. Third, considering that our participants were female with a high mean educational level, the results of this study may not be representative for men and those with lower educational level.

## Conclusion and future work

The present analyses sought to provide a micro-level perspective to what could be the phenotypic translation of the genetic and environmental liability to psychopathology. Although suggestive, this first study of differences between genetic liability and environmental strata did not show any evidence to support the hypothesis that genes and early adversity have an impact on emotional dynamics in daily life as measured by the current network methodology. In future work, the present exploration of the effect of genes and environment on the affective regulation network should be replicated using the sum scores positive and negative affect before expanding it to molecular genetic measures of risk such as polygenic risk scores or to the interaction between childhood trauma and genetic liability. For this, both general population studies as well as case-control studies should be designed to complete our understanding of the mechanisms underpinning mental disorders.

Furthermore, further testing of the basic network of AMS as an intermediate phenotype may also be of value as networks can be seen as ecologically valid phenotypes, complementary to categorical diagnostic phenotypes in genetic studies.

## Author contributions

LH contributed to the conception of the work, the analysis, interpretation of data for the work, and to drafting it. MD contributed to the conception of the work, the analysis, interpretation of data for the work and to drafting and revising it. SG and WV contributed to the interpretation of data for the work, drafting and revising it. MD, CD, and ET contributed to the acquisition of data for the work, and to revising it. JvO contributed to the conception of the work, the interpretation of data for the work, and to revising it.

### Conflict of interest statement

The authors declare that the research was conducted in the absence of any commercial or financial relationships that could be construed as a potential conflict of interest.

## Abbreviations

AMS: Affective Mental State
EFPTS: East Flanders Prospective Twin Study
ESM: Experience Sampling Method
NA: Negative Affect
PA: Positive Affect
SCL-90-R: Symptom Checklist-90-R
SD: Standard Deviation
MZ: Monozygotic
DZ: Dizygotic.
