# Supplementary figures and images for: Genetic and Environmental Influences on the Affective Regulation Network: A Prospective Experience Sampling Analysis
Source: Front Psychiatry. 2018 Nov 28;9:602. doi: 10.3389/fpsyt.2018.00602 (PMC6279878; doi:10.3389/fpsyt.2018.00602)

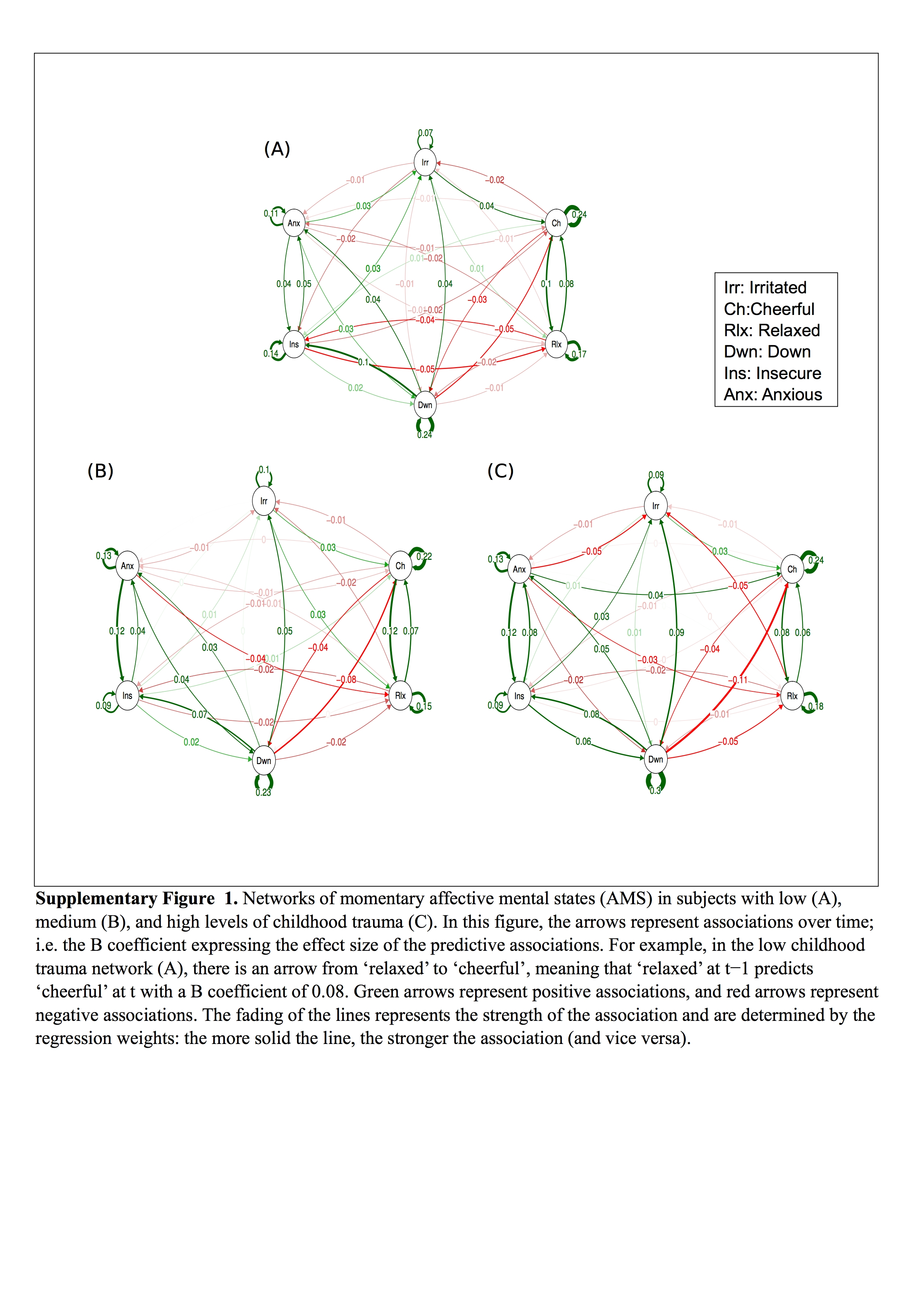

Supplement: Supplementary file 1 [file Image_1.TIFF]
