# Supplementary material for: Genetic and Environmental Influences on the Affective Regulation Network: A Prospective Experience Sampling Analysis
Source: Front Psychiatry. 2018 Nov 28;9:602. doi: 10.3389/fpsyt.2018.00602 (PMC6279878; doi:10.3389/fpsyt.2018.00602)
Supplement: Supplementary file 2 [file Image_2.pdf]

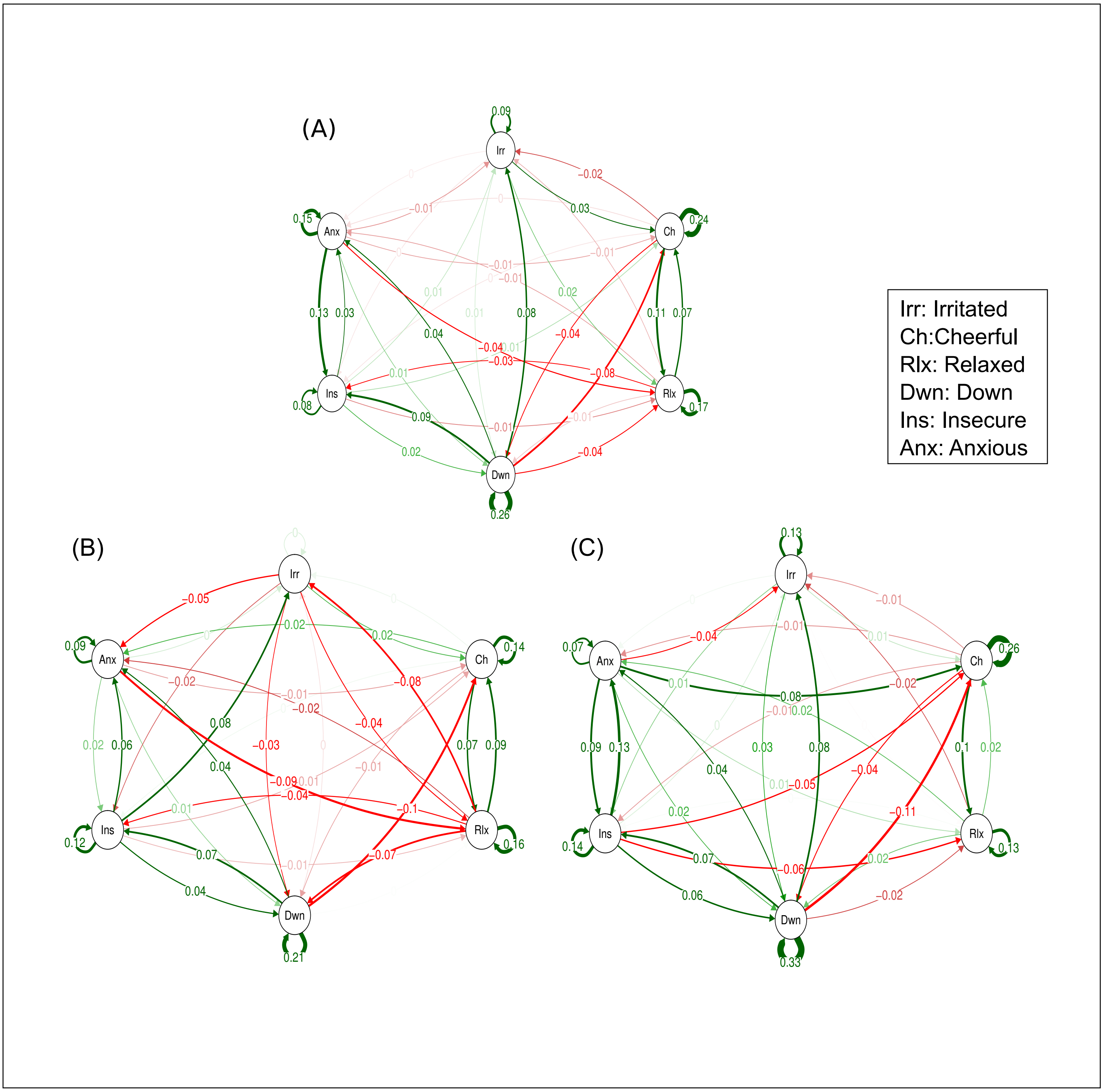

Supplementary Figure 2. Networks of momentary affective mental states (AMS) in participants with low (A), intermediate (B) and high genetic liability for psychopathology (C). In this figure, the arrows represent associations over time; i.e. the B coefficient expressing the effect size of the predictive associations. For example, in the low genetic liability network, there is an arrow from 'relaxed' to 'cheerful', meaning that 'relaxed' at t−1 predicts 'cheerful' at t with a B coefficient of 0.07. Green arrows represent positive associations, and red arrows represent negative associations. The fading of the lines represents the strength of the association and are determined by the regression weights: the more solid the line, the stronger the association (and vice versa).
